# Increased expression of α-methylacyl-coenzyme A racemase (AMACR; p504s) and p16 in distal hyperplastic polyps

**DOI:** 10.1186/1746-1596-8-178

**Published:** 2013-10-23

**Authors:** Nimet Dayi, Hideo A Baba, Kurt W Schmid, Klaus J Schmitz

**Affiliations:** 1 St. Barbara-Hospital, Barbarastrasse 1, 45964 Gladbeck, Germany; 2 Institute of Pathology and Neuropathology, University of Duisburg-Essen, Hufelandstrasse 55, 45122 Essen, Germany; 3 Institute of Pathology Recklinghausen, Mühlenstrasse 31, 45659 Recklinghausen, Germany

**Keywords:** AMACR, p16^Ink4a^, Hyperplastic polyp, Sessile serrated adenoma

## Abstract

**Background:**

Hyperplastic polyps (HP) and sessile serrated adenomas (SSA) share morphological similarities. In this immunohistochemical study we chose a panel of potential relevant and promising biomarkers including α-methylacyl-coenzyme A racemase (AMACR; p504s), which is involved in the degradation of branched chained fatty acids derivates, and analysed a cohort of HPs and SSAs in order to identify different immunophenotypes in relation to lesion localisation.

**Methods:**

154 specimen were carefully selected and a micro tissue array (TMA) was constructed. Immunohistochemistry of p16^Ink4a^, Ki67, α-methylacyl-coenzyme A racemase (AMACR; p504s), BRAF, CK 20, MLH1 and β-catenin was performed and and immunoexpression was compared among proximal and distal HPs as well as SSAs.

**Results:**

None of the markers revealed a differential expression among HPs and SSAs. However, the study demonstrates a significant overexpression of AMACR (p = 0.004) and p16^Ink4a^ (p = 0.028) in distal HPs compared to proximal HPs. In addition AMACR overexpression was associated with increased p16^Ink4a^ immunoexpression (p < 0.001).

**Conclusions:**

In this study we describe differential AMACR and p16^Ink4a^ in HPs in relation to their localisation. Distal HPs were characterized by AMACR and p16^Ink4a^ overexpression in contrast to proximal HPs, although morphological identically. Thus AMACR overexpression points towards a pathobiological relevance of the protein in distal HPs. In context of recently published data this suggest distal HPs as potential precursor lesions of certain adenoma subtypes. However, at this point of time this finding remains speculative and needs to be confirmed by further studies.

**Virtual slides:**

The virtual slide(s) for this article can be found here: http://www.diagnosticpathology.diagnomx.eu/vs/1836116001066768

## Background

Historically hyperplastic polyps have been considered benign and harmless lesions for several decades [1]. Meanwhile it is known, that besides of the classical adenoma-adenocarcinoma pathway described by Vogelstein [2], an additional (non-hereditary) pathways exists, called the serrated pathway [1,3-6] resulting in serrated lesions such as the sessile serrated adenoma (SSA) and serrated adenocacinoma. The serrated morphology of this pathway reflects the disorder in impaired apoptosis resulting in retention of epithelial cells at the base of the crypts.

Molecular key features of the classical adenoma-adenocarcinoma include KRAS and p53, whereas in the serrated pathway BRAF mutations appear to be an early event [2,7-9]. In addition this initial event is often followed by hypermethylation of CpG-island in gen promoters. As a result of hypermethylation e.g. MLH1 genes are silenced resulting in the well known and characteristic (sporadic) microsatellite instable phenotype and genotype, which often occurs in SSA and serrated adenocarcinoma [10]. Meanwhile several pathomorphogical criteria have been established, that allow a stricter separation of SSAs and hyperplastic polyps (HPs) [3,11]. However, clear separation of SSA and HP in individual cases is sometimes impossible. In addition, even HPs may harbour somatic BRAF mutations and an so-called CPG island methylator phenotype (CIMP) [12,13]. Thus a subset of HPs may be precursor lesions of SSAs.

The aim of this study was to analyse several immunohistochemical markers in order to 1) identify additional markers, that may allow a more clearly separation of HPs and SSAs and 2) to compare proximal and distal HPs and to work out potential different immunophenotypes of proximal versus distal HPs.

The antibodies used in this study were the following: 1) p16^Ink4a^, which is an important factor in carcinogenesis and was shown to be linked to oncogene-induced senescence in the serrated route to colon cancer [14]; 2) Ki67, which represents a established proliferation marker; 3) α-methylacyl-coenzyme A racemase (AMACR;p504s). AMACR is a mitochondrial and peroxisomal enzyme that is part of the degradation of branched chained fatty acid derivates. AMACR is essential for the completion of the β-oxidation pathway [15] and has recently been shown to upregulated in colorectal cancer and adenoma [16-18]; 4) BRAF, MLH1 and β-catenin as additional markers involved in the serrated adenocarcinoma carcinogenesis.

## Material and methods

### Specimen

Initially 891 biopsy specimen from 2009 to 2012 representing HPs and SSAs were retrieved from the files of the Institute of Pathology, Recklinghausen. The slides of these specimen were re-evaluated by the authors KJS and ND. All biopsies, that did not allow optimal evaluation of the complete lamina propria (including the base of the crypts) were excluded from further investigation (n = 737). In the remaining 154 specimen, lesion localisation was defined as proximal, if retrieved from coecum or ascending colon. Distal localization included specimen derived from descending colon, sigmoid or rectum. Table 1 depicts relevant clinical data of the study group.

**Table 1:** Clinical details of the study group

**Variables**	**Results**		
	**Proximal HPs**	**Distal HPs**	**SSAs**
*Gender*			
Male	21	39	18
Female	27	28	14
Mean age in years	65.45	62.70	65.35

### Tissuemicroarray (TMA) construction

SSA and HP areas were carefully selected and marked on the respective H&E (haematoxylin and eosin-stained) slides. TMA construction was performed as previously described [19]. The TMA included 154 specimen.

### Immunohistochemistry of TMA

The primary antibodies used as well as the technical details are summarized in Table 2.

**Table 2:** Antibodies used for immunohistochemistry

**Antibody**		**Pretreatment**	**Incubation**	**Detection**	**Dilution**	**Company**
AMACR	Monoclonal	98°C Hot water, pH 9.0,	15 min	Envision™	Ready-to-Use Kit	Dako, Glostrup, Denmark
p16Ink	Monoclonal, clone G175-405	98°C Hot water, pH 6.1,	20 min	Envision™	1:10	BD Biosciences, Heidelberg, Germany
Ki67	Monclonal, clone MIB-1	98°C Hot water, pH 9.0,	15 min	Envision™	Ready-to-Use Kit	Dako, Glostrup, Denmark
BRAF	Monoclonal, clone VE1	37°C Hot water, PH 9.0	30 min	Envision™	1:100	DCS Innovative Diagnostik, Hamburg, Germany
MLH1	Monoclonal, clone ES05	98°C, pH 9.0	20 min	Envision™	Ready-to-Use Kit	Dako, Glostrup, Denmark
Beta-catenin	Monoclonal	37°C water bath 30 min	20 min	Zytochem Plus HRP Polymer (Zytomed Systems, Berlin, Germany)	1:2000	BD Biosciences, Heidelberg, Germany
CK20	Monoclonal, Clone Ks20.8	98°C, pH 9.0	30 min	Envision™	Ready-to-Use Kit	Dako, Glostrup, Denmark

### Evaluation of immunohistochemistry

**AMACR**: AMACR expression was scored according to the intensity of solely basal crypt cytoplasmic staining pattern. AMACR negative (0): no basal staining; AMACR positive (1+): cytoplasmic immunostaining of basal located crypt epithelial cells, independent of the intensity.

**P16**: p16 expression was scored according to the intensity of solely basal crypt nuclear and cytoplasmic staining patterns. p16 negative: missing basal immunostaining; p16 positive: basal positive nuclear and cytoplasmic staining.

**Ki67**: Ki67 expression was scored according to the positivity of epithelial cells in the upper third of the crypts. Positive cells were counted.

**Cytokeratin 20**: all cases showed an upper crypt staining and scattered basal stained cells. Cases were scored according the Cytokeratin 20 staining pattern: usual pattern (staining confined to upper crypt zone) and unusual pattern (additional scattered positivity in the basal crypt zone).

**BRAF, MLH1 and β-catenin**: MLH1 immunostaining revealed no morphological differences among different groups (HP versus SSA) or different localizations (proximal vs. distal). A weak MLH1 staining was noticed in all specimen. BRAF immunostaining was weak to negative in all cases (although external control specimen containing BRAF mutations resulted in positive immunostaining). β-catenin immunostaining resulted in strong cytoplasmic and nuclear staining of the complete basal crypt zone of all specimen. BRAF, MLH1 and β-catenin were excluded from further analysis.

### Statistical analysis

All immunostainings were assessed by KJS and ND in a blind trial fashion. In case of disagreement, slides were re-evaluated by both investigators until agreement was reached. All data were converted to a PC and statistically analysed using SPSS version 20 for Macintosh (Statistical Package for Social Sciences, Chicago, IL, USA). Relationships between ordinal parameters were investigated using the two-tailed χ^2^ analysis. The relationship between categorical data (e.g. SSA versus HP) and numeric data (number of Ki67 positive cells) was determined using the Kruskal Wallis test.

## Results

Table 3 summarizes the amount of specimen included in this study and analysed for AMACR and p16 immunoexpression.

**Table 3:** Specimen analysed for AMACR and p16 immunoexpression

**AMACR**	**N**	**Localization**		**p16**	**N**	**Localization**	
	**106**	**Distal**	**Proximal**		**109**	**Distal**	**Proximal**
HPs	82	49	33	HPs	83	50	33
SSAs	24	11	13	SSAs	26	12	14

**Figure 1:**
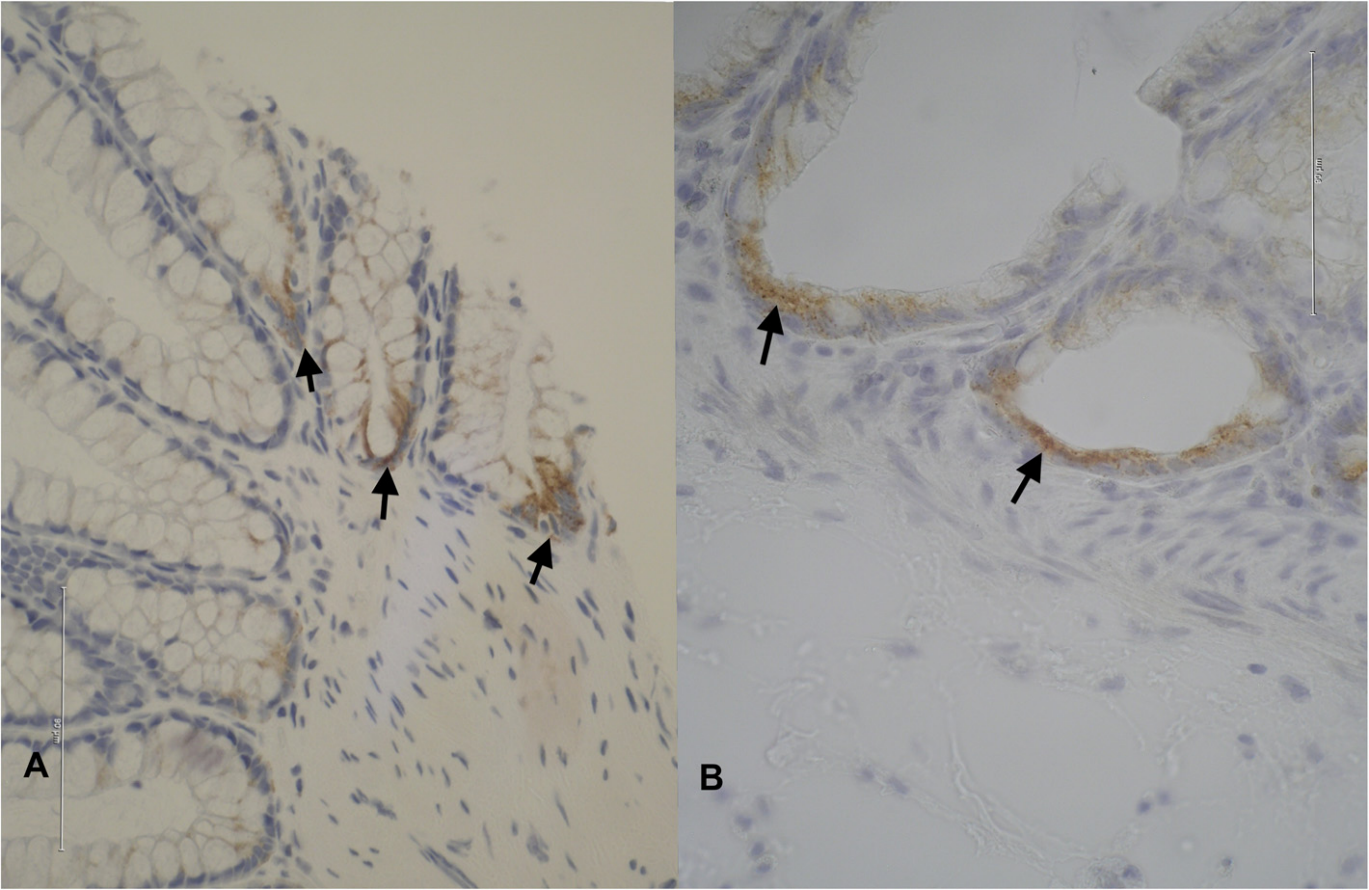
**On the left HP with strong basal AMACR immunoexpression.** On the right higher magnification of cytoplasmic granular AMACR expression at the base of the typically L-shaped, dilated SSA crypts (magnification × 400).

AMACR immunostaining was located in the cytoplasm of upper and basal crypt cells. Only basal location was analysed. Regarding the group of HPs, χ^2^ analysis showed, that distal HPs exhibited statistically significantly positive AMACR expression (p < 0.004; Table 4). Figure 1 demonstrates AMACR expression in SSA and HP.

**Table 4:** AMACR and p16 expression in proximal and distal HPs

	**AMACR (n = 82)**			**p16 (n = 83)**		
	**Negative**	**Positive**	**p-value**	**Negative**	**Positive**	**p-value**
Proximal HPs	28 (84,8%)	5 (15,2%)	0,004	31 (96,6%)	1 (3,1%)	0,028
Distal HPs	27 (55,1%)	22 (44,9%)		41 (80,4%)	10 (19,6%)	

Regarding the group of SSA, no statistically different AMACR immunoexpression could be detected among proximal and distal SSAs (p = 0.329). In addition a comparison of all SSAs versus all HPs revealed no differences in AMACR expression (p = 0.448).

**Figure 2:**
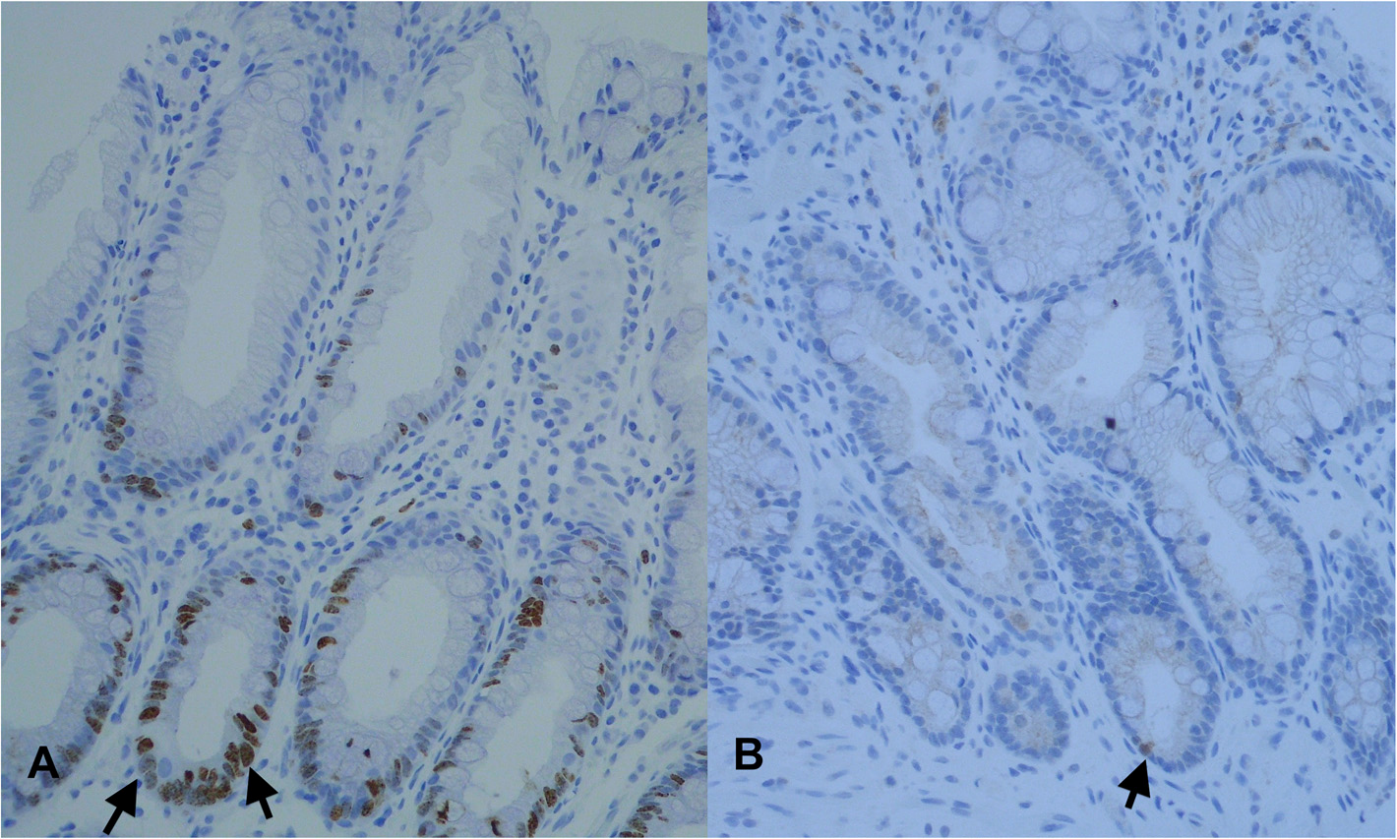
**On the left strong nuclear p16 expression in a distal hyperplastic polyp.** On the right missing to low p16 expression in a proximal HP (magnification × 200).

P16 immunostaining was located at the basal crypt zone in the cytoplasm and nucleus of crypt cells (Figure 2). If all HPs were compared to all SSAs no statistical difference in p16 expression was detected (p = 0.183). The analysis of proximal versus distal HPs demonstrated a significantly stronger p16 immunoexpression in distal HPs (p = 0.028; Table 4).

Further we analysed the association of AMACR and p16 expression within the group of HPs. There was a highly significant association of AMACR overexpression with p16 positivity in HPs (p < 0.001; Table 5).

**Table 5:** Association of AMACR and p16 in HPs

**HPs (n = 75)**	**p16 immunoexpression**		
	**Negative**	**Positive**	**p-value**
AMACR positive	49 (75%)	1 (9,1%)	<0.001
AMACR negative	27 (25%)	10 (90,9%)	

Ki67: Ki67 analysis of HPs versus SSAs and within the groups of HPs or SSAs revealed no significant results. There were no statistically significant differences between the mean Ki67 value in HPs versus SSAs. However the mean value of KI67 proliferating cells was higher in SSA compared to HP.

Cytokeratin 20: Cytokeratin 20 analysis of HPs versus SSAs and within the groups of HPs or SSAs revealed not significant results. There were no differences in the distribution of CK20 staining of the upper crypt zone and the basal zone.

MLH1: MLH1 analysis of HPs versus SSAs and within the groups of HPs or SSAs revealed not significant results. There were no differences in the distribution of MLH1 staining of the upper crypt zone and the basal zone.

Beta-catenin: analysis of HPs versus SSAs and within the groups of HPs or SSAs revealed not significant results. All cases revealed a cytoplasmic/nuclear staining at the basal crypt zone.

BRAF: All cases revealed a weak to negative staining without significant differences. All cases lacked positive staining results, although HPs frequently demonstrate BRAF mutations and despite of positive external study controls. Thus BRAF immunohistochemistry in this study is not a reliable tool to detect BRAF mutations in HPs or SSAs.

## Discussion

This study on a cohort of HPs and SSAs demonstrates, that HPs exhibit different immunophenotypes regarding to their localization (proximal vs. distal). The Immunohistochemical analysis included the following markers: AMACR, p16^Ink^, CK20, Ki67, BRAF, β-catenin and MLH1. We focussed on the expression of AMACR and p16^Ink^ in HPs and SSAs with respect on lesion localisation since analysis of CK20, Ki67, BRAF, β-catenin and MLH1 either lacked significant results or resulted in unreliable data. All parameters analysed are involved in the serrated adenocarcinoma pathway. Besides the well known AMACR overexpression in prostate cancer [20], overexpression was also demonstrated in colorectal cancer [21]. Moreover dysplastic epithelial cells in Barrett Oesophagus were shown to be associated with elevated AMACR levels [22].

Likewise SSAs, HPs have been shown to harbour specific genetic alterations such as somatic BRAF mutations and CPG island methylator phenotype (CIMP) [12,13]. This suggests the progression of a subset of HPs towards SSAs. Thus one of our study aims was to identify potential biomarkers, that may allow more precise separation of HPs and SSAs and identify a subset of HPs that might qualify as precursor lesions of SSAs. However, the analysed biomarkers did not provide additional diagnostic information allowing a more distinct differentiation of HPs and SSAs. Recently Ki67 counting and distribution was shown to be different in HPs versus SSAs, with higher Ki67 values in SSAs [23]. However, in our study we could not confirm this finding. This might be due to the fact, that in our study only a small number of SSAs were analysed. In addition in the study by Fujiimori et al. Ki67 evaluation was calculated using automated image processing software, whereas this was not the case in the present study.

Furthermore this study describes a significant overexpression of AMACR in left-sided distal HPs when compared to right-sided proximal HPs. Thus although morphological identically these lesions exhibit a varying AMACR immunophenotype. It is unlikely, that these distal HPs are at higher risk to progress to SSAs, since SSAs are typically located proximal in the right colon. Given the known function of AMACR regarding fat metabolism, it may be possible, that in the distal parts of the colon the mucosa is exposed to an increased amount of branched-chain fatty acids and fatty acid derivates.

Another possible explanation would be a specific supportive role of AMACR in the development of precursor lesions in the colorectal cancer carcinogenesis. This hypothesis is supported by recent data demonstrating increased AMACR expression in high grade dysplasia compared to low grade dysplasia in conventional adenomas [18].

Until now only few studies focussed on AMACR expression in gastrointestinal tumours. In a recent study on 1315 colorectal cancers AMACR overexpression was found to be associated with left-side tumour localisation in colorectal cancer [16]. In detail, AMACR elevation was significantly associated with higher tumour differentiation grade (G1 and G2) and advanced tumour stage. In addition high AMACR expression levels were related to a tubular phenotype and less often to mucinous or signet cell carcinomas. These results presented by Marx et al. point towards a relevant role of AMACR expression at least in a subgroup of colorectal cancer and implicate a linkage of AMACR expression and site-related differences in metabolism/exposure to fatty acids.

In the present study in addition to differential expression of AMACR in HPs, we were able to detect significant differences in the expression of p16^Ink4^ in proximal and distal HPs. Similar to AMACR, p16^Ink4^ was overexpressed in distal HPs and significantly associated with AMACR overexpression. p16^Ink4^ is a well characterized protein with an important role in oncogene-induced cell aging [24,25]. Upregulation of p16^Ink4a^ was recently shown to function as an senescence barrier in the serrated route to colon cancer [14].

Taken together the overexpression of AMACR and p16^Ink^ in distal HPs points toward a pathophysiological relevance of both these proteins. It is now necessary to identify in which processes AMACR and p16^Ink^ in distal HPs are involved and if their dysregulation is implicated in the development of certain subgroups of adenomas.

Highly interesting in this context is a very recent study from Zhang et al. that analyses AMACR expression in normal mucosa, adenoma and colorectal carcinoma both on immunohistochemical and genetic level [17].

Zhang et al. reported AMACR negativity in normal colonic mucosa and tubular adenoma with low grade and intermediate dysplasia as well as in poorly differentiated carcinoma. In contrast AMACR overexpression was found in villous adenoma and high and moderately differentiated colon cancer. These results are in concordance with those of Marx et al. Using Laser-capture-microdissection Zhang et al. discovered different deletions in the AMACR promotor CpG Island, depending upon the underlying tissue: In normal colonic glands and tubular adenomas with low AMACR expression they detected a somatic double-deletion at CG3 and CG10, that was absent in villous adenomas and all colon cancers with variable AMACR levels. In contrast they identified a high prevalence (89%) of deletion of CG12-16 in moderately differentiated colon cancers with strong AMACR overexpression, whereas these deletions existed in only 14% of poorly differentiated colon cancer. This deletion of CG12-16 was shown to be a constitutional allele with a frequency of 43% in the general population.

Taken together, AMACR protein expression may be regulated by somatic or constitutional genetic alterations in AMACR Promoter CPG islands. Keeping the above mentioned findings in mind, it is now tempting to speculate, that distal HPs with increased AMACR expression might constitute precursor lesions of a pathway leading to the development of villous adenomas and/or subtypes of low grade colorectal cancers.

## Conclusions

This study demonstrates differential expression of AMACR and p16 in morphologically similar looking HPs in relation to their localisation. Distal located HPs exhibit AMACR and p16 overexpression compared to proximal HPs. The reasons and pathobiologically significance of this varying immunophenotype needs be elucidated in further studies.

## Competing interests

The authors declare that they have no competing interests.

## Authors’ contributions

ND and KJS drafted the manuscript, ND analysed Immunohistochemical data and performed TMA construction and data collection. HAB and KWS participated in the design of the study, have made substantial contributions to conception and design as was data analysis and have given final approval of the version to be published. KJS has performed statistical analysis. All authors read and approved the final manuscript.
